# *In silico* Analysis of Human Telomerase Reverse Transcriptase (*hTERT*) Gene: Identification of a Distant Homolog of Melanoma Antigen Family Gene (MAGE)

**DOI:** 10.4137/cin.s3392

**Published:** 2009-11-24

**Authors:** Ruhul Amin, Jesmin, Hasan Jamil, M. Anwar Hossain

**Affiliations:** 1 Department of Microbiology, University of Dhaka, Dhaka 1000, Bangladesh; 2 Department of Genetic Engineering and Biotechnology, University of Dhaka, Dhaka 1000, Bangladesh; 3 Department of Computer Science, Wayne State University, Michigan, USA. Email: anwarhossain@hotmail.com or jesmin@univdhaka.edu

**Keywords:** telomerase, melanoma antigen, gene prediction, EST, ORF

## Abstract

Melanoma antigen family (MAGE) genes are widely expressed in various tumor types but silent in normal cells except germ-line cells lacking human leukocyte antigen (HLA) expression. Over 25 MAGE genes have been identified in different tissues, mostly located in Xq28 of human chromosome and some of them in chromosome 3 and 15, containing either single or multiple-exons. This *in silico* study predicted the genes on *hTERT* location and identified a distant relative of MAGE gene located on chromosome 5. The study identified a single exon coding ~850 residues polypeptide sharing ~30% homology with Macfa-MAGE E1 and hMAGE-E1. dbEST search of the predicted transcript matches 5′ and 3′ flanking ESTs. The predicted protein showed sequence homology within the MAGE homology domain 2 (MHD2). UCSC genome annotation of CpG Island around the coding region reveals that this gene could be silent by methylation. Affymetrix all-exon track indicates the gene could be expressed in different tissues particularly in cancer cells as they widely undergo a genome wide demethylation process.

## Introduction

Human telomerase, a cellular reverse transcriptase, is a ribonucleoprotein enzyme that catalyzes the synthesis and extension of telomeric DNA.^1^ Telomerase activity appears to be associated with cell immortalization and malignant progression.^2^ Usually human telomerase is found in hemopoietic and in germ cells but not in normal somatic cells. Active telomerase is one of the key factors that enable malignant cells to proliferate indefinitely.^3^ However, molecular mechanisms triggering various telomerase activities still remain elusive.

In this post-genomic era, *in silico* techniques for gene finding process or identifying the location of the protein-coding regions (ORF), within uncharacterized genomic DNA sequences, constitute a central issue in the field of bioinformatics^4^ and are of much interest to biologists. A number of computational techniques for the prediction of distinctive features of protein-coding regions have been proposed along with the standard molecular methods. In general, the two main approaches of structural gene prediction are *intrinsic* (based on statistical properties of exons, splice sites, and other signals) and *extrinsic* (based on homology with known genes).^5^

In this study, the human chromosome 5p13.1–p15.33 region containing the telomerase (*hTERT*) gene was investigated by using both *intrinsic* and *extrinsic* method of gene prediction. In addition to *hTERT* ORF, two additional ORFs named *gene2* and *gene3* were identified. Interestingly, predicted *gene2* revealed significant sequence homology with human tumor specific antigen, melanoma antigen family gene (MAGE), E1.

## Materials and Methods

Complete sequence of human telomerase reverse transcriptase (*hTERT*) was retrieved from NCBI (gi:82399156, Accession no. DQ264729.1).

### *In silico* identification of ORFs

The coding sequences of *hTERT* were identified using NCBI’s ORF Finder (http://www.ncbi.nlm.nih.gov/gorf). The sequence (DQ264729.1) was further analyzed using various *ab initio* gene finding programs (GENSCAN, FGENESH and AUGUSTUS) and by comparative gene prediction (TWINSCAN) methods. The genomic location of the *hTERT* was studied using the UCSC Genome browser (http://genome.ucsc.edu/cgibin/hgGateway). This browser was also used to identify the CpG Island track and EST’s around the predicted ORF. The 5′-UTR of the predicted transcription start site (TSS), the start codon and the 3′-UTR, 1000 bp downstream from the stop codon were searched using BLASTn, against the EST database (dbEST).

### Homology study of the predicted ORFs

The homologous sequences of the predicted genes were identified from successive iterations using PSI-BLAST. Multiple sequences were aligned using ClustalW (1.83) (http://www.ebi.ac.uk/tools/clustalw). Secondary structure of the predicted *gene2* was analyzed using the program Hierarchical Neural Network (HNN: http://www.expasy.org/tools/). The repeated pattern motifs were analyzed using the program Rapid Automatic Detection and Alignment of Repeats RADAR (http://ebi.ac.uk/radar/).

### Comparative genomics analysis of the predicted *gene2*

Global alignment of the coding sequence of *gene2* with Chimpanzee (*Pan troglodytes*) and Orangutan (*Pongo pygmaeus-abelii*) genomic sequence was performed with the program AVID using a window size of 100 bp and a conservation level of 70%. Results were viewed with the program VISTA.^6^ Finally, the nucleotide sequence of the Chimpanzee *TERT* (*Pan troglodytes* chromosome 5 genomic contig, reference assembly, Accession no. NW_001235370, region: 211936–253254) was further analysed by using GENSCAN to confirm the presence of conserved *gene2* in Chimpanzee genome.

### Prediction of the function of the *gene2*

The function of *gene2* was predicted using two protein function prediction program PFP^7^ and SVMProt.^8^ For comparative analysis, function of MAGE-E1 was also predicted using these two programs.

## Result and Discussion

Table 1 showed the gene prediction analysis of *hTERT* by different programs. GENSCAN^9^ had predicted 4 genes within the same *hTERT* location. Interestingly, apart from the known *hTERT* splice variant (*gene1* and *gene4* on the +strand), this program also predicted two additional genes in the reverse strand, namely *gene2* (9155–5064 bp) and *gene3* (16386–12458 bp) both of which consists of 3 exons. AUGUSTUS^10^ also identified two genes in the reverse strand at a slightly different location-*gene2* (7869–5617 bp) consists of a single exon whereas *gene3* (14225–12469 bp) consists of two exons. The predicted genes in the reverse strand were confirmed by the FGENESH and ORF Finder. Homology-based program TWINSCAN^11^ also predicted two reverse strand genes in the same way as FGENESH and ORF Finder (Table 1).

**Figure 1:**
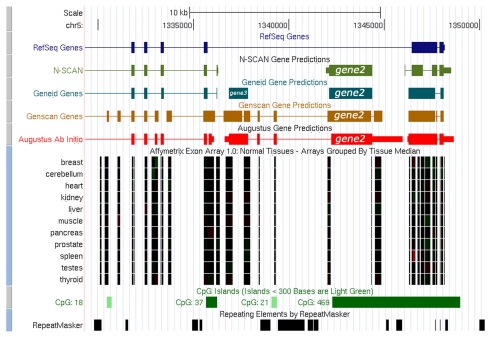
UCSC Genome Browser analysis showing the location of the predicted genes. Affymetrix All Exon Chip-Array tracks are used for tissue specific gene expression. CpG Island is also observed around the predicted gene.

In UCSC genome browser, two different gene prediction tracks NSCAN and GeneID also predicted the location of the *gene2* and *gene3* (Fig. 1). Tissue-specific expression pattern of the predicted genes was hypothesized by observing Affymetrix all-exon track. The genome browser also predicted the CpG island around the predicted genes. A good number of ESTs were identified at the 5′ and 3′ flanking regions for both the predicted *gene2* and *gene3*. The predicted ESTs showed different expression profiles in different tissue types. Interestingly for the predicted *gene2* it has been found that in some tissue types the gene is expressed in developmental stages and in others expressed in different cancer cell lines (Fig. 1). Similar patterns were also observed for the predicted *gene3* and revealed that it shares good 5′ flanking region (~99% identity) with the clone collection from IMAGE cDNA, mRNA database sequences (Supplementary data).

**Figure 2:**
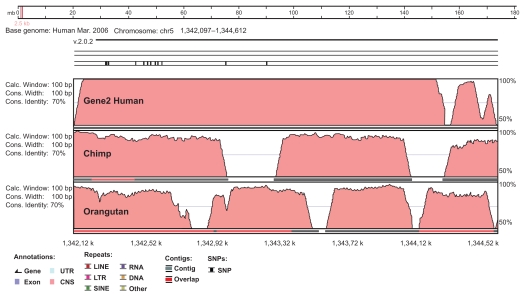
Conservation of *gene2* in Chimpanzee (*Pan troglodytes*) and orangutan (*Pongo pygmaeus-abelii*) on VISTA browser.

VISTA plot of the AVID alignment (Fig. 2) indicated that majority of the region in *gene2* is highly conserved (>92% identity) in Chimpanzee and Orangutan genome. No larger repeats (LINE, SINE, and LTR) were observed within that region. Further GENSCAN analysis of the corresponding TERT region on Chimpanzee chromosome 5 indicated that this region also contain a putative gene encoding 748 amino acids protein which is very much similar (82% identity and 84% similarity) to the predicted *gene2* of *hTERT* region (Supplementary data). Identification of the blocks of genes with evolutionary conserved order in multiple genomes is an important issue in comparative genomics. These synteny blocks help in tracing back the evolution of genomes in terms of rearrangement event. Presence of conserved blocks of genes in multiple genomes may indicate functional relatedness of their products or presence of functionally important conserved non-coding regions.^12^ Through comparative genomics analysis of our predicted *gene2*, we have found that this gene is conserved in different genomes in terms of gene order.

**Figure 3:**
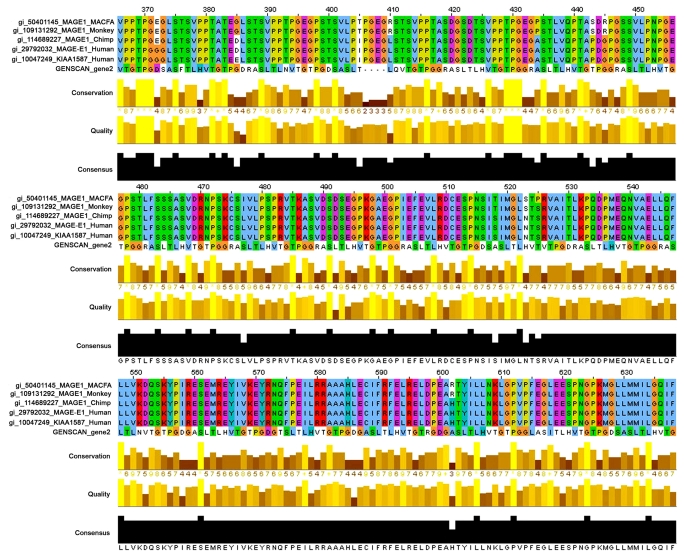
Global alignment of *gene2* with MAGE homology domain (MHD).

BLAST results indicated that *gene2* shared some homology (~38%–41%) with the MAGE E1, a member of type II melanoma antigen family (Table 2). MAGE family is a large family which comprises over 25 members identified in human. Most members of the MAGE family are clustered at the Xq28 region of human chromosome.^13^ The overall structure of MAGE-E1, and Macfa MAGE-E1 are larger proteins with extended N or C-termini. The N-terminal domain of MAGE-E1, contains a loosely conserved region of ~220 amino acids, termed MHD2 domain.^14^ Global alignment in ClustalW showed loosely conserved regions for both amino-terminal and carboxy-terminal domain of *gene2* with human MAGE family E1 (accession no. AAH50588.1) and MAGE1_Macfa, Melanoma associated antigenE1 (accession no. Q9BE18) (Fig. 3).

Identification of the novel members of a large protein family is very difficult as the similarity searching programs are designed to highlight the most similar sequences. As a result, about 5% of the novel protein family members may remain unrecognized.^15^ In an example by Retief et al,^15^ the large family of known glutathione transferase proteins was first subjected to multiple sequence alignment, and a phylogenetic tree was made by distance methods to identify classes of proteins within the family. These proteins represented a broad range of phylogenetic context and included classes with sometimes less than 20% identity.^16^ Thus, in spite of having ~40% sequence homology with MAGE-E1, predicted *gene2* may be a novel member of melanoma antigen family (MAGE).

Expression pattern of different members of the MAGE family genes are different and tissue specific, some encodes tumor specific antigens and some are expressed in normal cells.^17^,^18^ Besides, co-expression pattern of some tumour associated antigen (TAA) including 5 MAGE-A genes with human telomerase reverse transcriptase was observed in non-small cell lung carcinoma (NSCLC) such as adenocarcinoma, squamous cell carcinoma and bronchiolocarinomas.^19^ 5′ flanking EST analysis of predicted *gene2* revealed that most of the ESTs are expressed in several cancer cells. As human telomerase activity is observed only in germline and cancer cells^2^ and its co-expression patterns was observed with some MAGE gene, our predicted *gene2* may be co-expressed with telomerase in cancer cells.

Transcriptional regulation of MAGE gene family is dependent on various factors. Promoter methylation is one of these factors. Methylated CpG point in the promoter region is responsible for transcriptional silencing of the MAGE gene.^20^ Demethylation of the MAGE1 promoter appears to be sufficient to activate this gene in tumor cell lines.^21^ From UCSC genome browser, we have observed the presence of CpG island around our predicted *gene2*. That’s why, it can be inferred that the hypermethylation of this CpG island is responsible for silencing of *gene2* in normal cells and this gene may be activated through transformation dependent loss of DNA methylation in cancer cells.^22^–^24^

Repeated insertion appears to have played a major role during evolution of MAGE family. For instance, the long C-terminal domain of MAGE-D3 was most probably formed by serial duplications of decapeptide repeats. Also the N-terminal domains of MAGE-C1 and MAGE-D1, are highly repetitive, must have undergone sequential duplication events.^13^,^25^ 29 repeated short peptides containing ~12 amino acids were identified in *gene2* protein by using the program RADAR^26^ (Table 3). Although, function of these repeats is unknown, it revealed that *gene2* may be evolved from repeated insertion as found in MAGE family. To compare the repetitive pattern of gene2 with MAGE family protein, we have analyzed the repeats of MAGE-E1, MAGE-D1 and MAGE-D3 proteins (supplementary data). It was found that the repeats in these MAGE family members are not identical and in some cases several variations in amino acids are found. The pattern of MAGE-D1 repeat is WQXPXX^14^ which is completely different from MAGE-D and MAGE-E1. However, these MAGE family members are univocal in that sense that they all contain repeats (may be identical or different). On the other hand, our predicted *gene2* contains TPG repeat which is found at several position of MAGE-E1.

**Figure 4:**
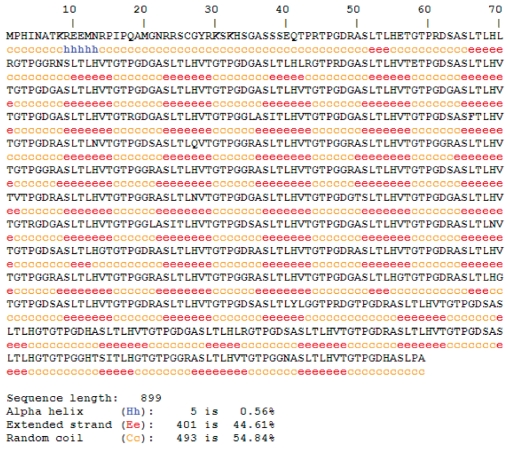
Secondary structure prediction of *gene2* using HNN.

The predicted secondary structure of *gene2* showed more extensive β-strands (~44%) and coil region (~55%) distributed along the sequence (Fig. 4). From the secondary structure of entire MAGE-E1, it was found that N-terminal MHD2 domain contains extensive coil and some β-stranded region but C-terminal MHD1 domain contains more α-helical regions along with some β-stranded regions. We have further analyzed the secondary structure pattern of repeats on MAGE-E1, MAGE-D1 and MAGE-D3 (supplementary data). Overall secondary structure pattern within the repeat region of these three family members was coil followed by β-strands but no α-helices which are very much likely to the secondary structure pattern of *gene2* repeats although the amino acid composition of the repeats is slightly different.

To understand the functional association between *gene2* and MAGE-E1, we have analyzed the protein function prediction result using two programs SVMProt and PFP. SVMProt results indicated that both *gene2* and MAGE-E1 belong to Zinc-binding protein family (Table 4). PFP result classified the function of protein according to the GO annotation categories. In the biological process categories, MAGE-E1 belongs to glia cell migration, nerve growth factor receptor signaling pathway, neuronal migration, brain development etc (Table 5) and *gene2* belongs to neurogenesis whose specific outcome is the progression of nervous tissue over time, from its formation to its mature state which is very much likely to MAGE-E1. In the molecular function categories, *gene2* matches with MAGE-E1 in exo-alpha-sialidase activity and inositol-polyphosphate 5-phosphatase activity. In the cellular component categories, both *gene2* and MAGE-E1 belong to dendrite, dystrophin-associated glycoprotein complex, actin cytoskeleton and nuclear region. Some differences in the prediction categories between *gene2* and MAGE-E1 are observed. This may be due to the limited functional analysis on MAGE family protein and still now we don’t know enough about the expression and function of MAGE family proteins.^14^ From these analyses, it can be inferred that as a distant member, there are some functional alliance of *gene2* with MAGE family protein.

However, low amino acid sequence homology was found for *gene3* protein. But it showed high homology (99% identity) with some human ESTs (Supplementary data). For this reason, we are predicting that this gene is a novel one or it may have functions in regulation rather than coding.

## Conclusion

Even though these predicted genes should be further characterized by laboratory means before their existence can be conclusively affirmed, the results presented in this study suggested and identified the location of a structurally similar gene of MAGE family on human chromosome 5. The findings can provide new insight in transcriptional activation of novel genes during malignant melanoma.

## Figures and Tables

**Table 1:** Summary of the gene prediction analysis result of *gene2* and *gene3*.

Gene Prediction Program	Predicted Genes	Strand	Features	Start	End
**GENSCAN**	*gene2*	Complement	Promoter	9515	9476
			Initial exon	9155	9102
			Internal exon	7861	5671
			Terminal exon	5518	5064
			Poly A site	4718	4713
	*gene3*	Complement	Promoter	16734	16116
			Initial exon	16386	16116
			Internal exon	14465	13849
			Terminal exon	13831	12458
			Poly A site	9749	9744
**AUGUSTUS**	*gene2*	Complement	CDS	7869	5617
	*gene3*	Complement	CDS-1	14225	13860
			CDS-2	13689	12469
**FGENESH**	*gene2*	Complement	TSS	9618	9618
			Exon-1	7858	5606
			Poly A site	4723	4723
	*gene3*	Complement	TSS	16699	16699
			Exon-1	16195	16116
			Exon-2	13907	12458
			Poly A site	11428	11428
**TWINSCAN**	*gene2*	Complement	Exon-1	7858	5606
	*gene3*	Complement	Initial exon	16386	16116
			Internal exon	14465	13849
			Terminal exon	13831	12458
**ORF Finder**	*gene2*	Complement	ORF in Frame-2	7858	5606
	*gene3*	Complement	ORF in Frame-3	13122	12106

**Table 2:** Summary of the homologous sequences of predicted *gene2.*

GI No.	Accession No.	Name of the protein	Length (aa)	Identity (%)	Similarity (%)	E-value
67604778	XP_666642.1	Cell surface protein that may regulate cell wall beta-glucan synthesis and bud site selection *[Cryptosporidium hominis* TU502]	999	27.76	48.53	2e-28
88602575	YP_502753.1	Mucin 2, intestinal/*trac heal[Methanospirillum hungatei* JF-1]	2353	27.69	36.69	6e-26
66363458	XP_628695.1	Serine/threonine rich low complexity protein *[Cryptosporidium parvum* Iowa II]	951	27.25	51.0	8e-23
71402846	XP_804287.1	Cellulosomal scaffoldin anchoring protein *[Trypanosoma cruzi* strain CL Brener]	928	40.67	44.33	2e-08
114771991	ZP_01449380.1	Fibronectin type III domain protein [alpha proteobacterium HTCC2255]	2282	25.0	38.33	2e-08
52144448	YP_082380.1	Collagen-binding surface protein *[Bacillus cereus* E33L]	913	25.38	30.85	4e-08
50401145	Q9BE18	Melanoma-associated antigen E1 (MAGE-E1 antigen) Macfa	957	28.0	41.0	5e-08
89095693	ZP_01168587.1	RTX toxins and related Ca2+-binding protein *[Bacillus sp.* NRRL B-14911]	1415	23.33	38.0	1e-04
29792032	AAH50588.1	Melanoma antigen family E, 1 *[Homo sapiens]*	957	28.0	41.67	6e-04
118716717	ZP_01569254.1	Hemagluttinin domain protein [*Burkholderia multivorans* ATCC 17616]	1487	25.4	50.0	0.004

**Table 3:**
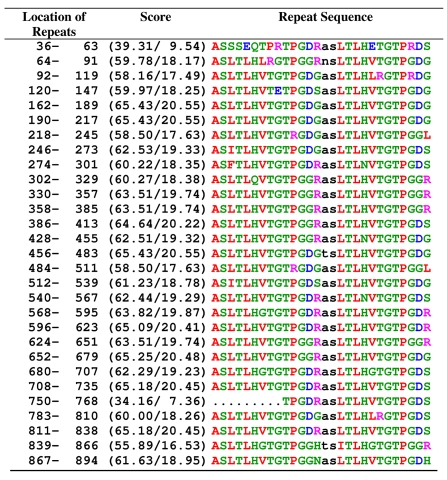
Repeated sequence motif of the predicted *gene2* analyzed by RADAR.

**Table 4:** Comparative function prediction of MAGE-E1 and *gene2* using SVMProt.

MAGE-E1		*Gene2*	
Function	P Value (%)	Function	P Value (%)
Zinc-binding	73.8	Zinc-binding	58.6
Nuclear receptors	65.4	Metal-binding	58.6
		EC 3.4 Hydrolases—Acting on peptide bonds (Peptidases)	58.6
		Calcium-binding	58.6
		TC 1.B. Channels/Pores—Beta-Barrel porins	58.6

**Table 5:** Comparative function prediction of MAGE-E1 and *Gene2* using PFP.

MAGE-E1		*Gene2*	
**Biological process**	**Score**	**Biological process**	**Score**
GO.0008347 glia cell migration	2907203.66	GO.0009405 pathogenesis	760364.22
GO.0048011 nerve growth factor receptor signaling pathway	1164048.92	GO.0008380 RNA splicing	539421.38
GO.0019233 perception of pain	961041.64	GO.0000398 nuclear mRNA splicing, via spliceosome	380866.18
GO.0001764 neuronal migration	868699.27	GO.0007520 myoblast fusion	365931.67
GO.0042060 wound healing	500313.41	GO.0007399 neurogenesis	222373.08
GO.0000074 regulation of cell cycle	473884.91	GO.0006512 ubiquitin cycle	205861.13
GO.0001558 regulation of cell growth	367704.56	GO.0016574 histone ubiquitination	205248.68
GO.0007585 respiratory gaseous exchange	316119.23	GO.0006816 calcium ion transport	187647.36
GO.0009062 fatty acid catabolism	263724.05	GO.0008104 protein localization	177700.20
GO.0007420 brain development	263045.24	GO.0042110 T-cell activation	171905.85
**Molecular function**	**Score**	**Molecular function**	**Score**
GO.0043015 gamma-tubulin binding	550081.62	GO.0004308 exo-alpha-sialidase activity	285616.06
GO.0016798 hydrolase activity, acting on glycosyl bonds	462333.96	GO.0046982 protein heterodimerization activity	258717.67
GO.0004308 exo-alpha-sialidase activity	451789.12	GO.0008332 low voltage-gated calcium channel activity	205987.38
GO.0004445 inositol-polyphosphate 5-phosphatase activity	222224.77	GO.0016874 ligase activity	190741.51
GO.0005515 protein binding	154771.23	GO.0030215 semaphorin receptor binding	161852.12
GO.0004674 protein serine/threonine kinase activity	132065.84	GO.0008168 methyltransferase activity	125300.40
GO.0003968 RNA-directed RNA polymerase activity	126483.06	GO.0004568 chitinase activity	112219.51
GO.0008289 lipid binding	119001.75	GO.0042809 vitamin D receptor binding	90949.14
GO.0017016 Ras interactor activity	106852.28	GO.0004842 ubiquitin- protein ligase activity	87944.78
GO.0004806 triacylglycerol lipase activity	105189.12	GO.0004445 inositol-polyphosphate 5-phosphatase activity	73545.05
**Cellular component**	**Score**	**Cellular component**	**Score**
GO.0045211 postsynaptic membrane	1992254.90	GO.0030425 dendrite	561550.58
GO.0030425 dendrite	811128.08	GO.0009986 cell surface	229447.45
GO.0016010 dystrophin-associated glycoprotein complex	493386.26	GO.0005891 voltage-gated calcium channel complex	220997.24
GO.0005813 centrosome	380800.79	GO.0005681 spliceosome complex	151073.10
GO.0048471 perinuclear region	276406.16	GO.0015629 actin cytoskeleton	125368.18
GO.0015629 actin cytoskeleton	178410.28	GO.0005737 cytoplasm	63162.21
GO.0046581 intercellular canaliculus	172680.03	GO.0016010 dystrophin-associated glycoprotein complex	58828.49
GO.0005634 nucleus	150265.62	GO.0005615 extracellular space	53737.16
GO.0005925 focal adhesion	136189.60	GO.0005643 nuclear pore	47225.56
